# Clinical Multigene Panel Sequencing Identifies Distinct Mutational Association Patterns in Metastatic Colorectal Cancer

**DOI:** 10.3389/fonc.2020.00560

**Published:** 2020-05-07

**Authors:** Francesca Belardinilli, Carlo Capalbo, Umberto Malapelle, Pasquale Pisapia, Domenico Raimondo, Edoardo Milanetti, Mahdavian Yasaman, Carlotta Liccardi, Paola Paci, Pasquale Sibilio, Francesco Pepe, Caterina Bonfiglio, Silvia Mezi, Valentina Magri, Anna Coppa, Arianna Nicolussi, Angela Gradilone, Marialaura Petroni, Stefano Di Giulio, Francesca Fabretti, Paola Infante, Sonia Coni, Gianluca Canettieri, Giancarlo Troncone, Giuseppe Giannini

**Affiliations:** ^1^Department of Molecular Medicine, University La Sapienza, Rome, Italy; ^2^Department of Public Health, University Federico II, Naples, Italy; ^3^Department of Physics, University La Sapienza, Rome, Italy; ^4^Institute for System Analysis and Computer Science “Antonio Ruberti”, National Research Council, Rome, Italy; ^5^National Institute of Gastroenterology-Research Hospital, IRCCS “S. de Bellis”, Bari, Italy; ^6^Department of Radiological Oncological and Pathological Sciences, University La Sapienza, Rome, Italy; ^7^Department of Surgery Pietro Valdoni, Faculty of Medicine and Dentistry, Sapienza University of Rome, Rome, Italy; ^8^Department of Experimental Medicine, University La Sapienza, Rome, Italy; ^9^Center for Life Nano Science@Sapienza, Istituto Italiano di Tecnologia, Rome, Italy; ^10^Pasteur Institute-Cenci Bolognetti Foundation, Rome, Italy

**Keywords:** mCRC, NGS, molecular stratification, mutation, genes

## Abstract

Extensive molecular characterization of human colorectal cancer (CRC) via Next Generation Sequencing (NGS) indicated that genetic or epigenetic dysregulation of a relevant, but limited, number of molecular pathways typically occurs in this tumor. The molecular picture of the disease is significantly complicated by the frequent occurrence of individually rare genetic aberrations, which expand tumor heterogeneity. Inter- and intratumor molecular heterogeneity is very likely responsible for the remarkable individual variability in the response to conventional and target-driven first-line therapies, in metastatic CRC (mCRC) patients, whose median overall survival remains unsatisfactory. Implementation of an extensive molecular characterization of mCRC in the clinical routine does not yet appear feasible on a large scale, while multigene panel sequencing of most commonly mutated oncogene/oncosuppressor hotspots is more easily achievable. Here, we report that clinical multigene panel sequencing performed for anti-EGFR therapy predictive purposes in 639 formalin-fixed paraffin-embedded (FFPE) mCRC specimens revealed previously unknown pairwise mutation associations and a high proportion of cases carrying actionable gene mutations. Most importantly, a simple principal component analysis directed the delineation of a new molecular stratification of mCRC patients in eight groups characterized by non-random, specific mutational association patterns (MAPs), aggregating samples with similar biology. These data were validated on a The Cancer Genome Atlas (TCGA) CRC dataset. The proposed stratification may provide great opportunities to direct more informed therapeutic decisions in the majority of mCRC cases.

## Introduction

Colorectal carcinoma (CRC) is one of the most commonly diagnosed cancers worldwide (1, 2). A large proportion of patients develop distant metastasis, which contributes to the high mortality reported for this tumor. With the current standard approaches, the 5-year survival rate for metastatic CRC (mCRC) is about 13% (1–3). These oxaliplatin or irinotecan-based chemotherapy regimens allow a median overall survival (OS) of about 18–20 months (4, 5). Survival rates can be significantly improved by a “triplet” approach consisting of 5-FU, oxaliplatin, and irinotecan chemotherapy (6) and/or by the addition of targeted drugs, such as monoclonal antibodies directed against angiogenesis or EGFR pathway (7). Nonetheless, median OS for mCRC rarely exceeds 30–36 months (8–10). Unfortunately, individual responses to these therapeutic approaches may be dramatically different from patient to patient reflecting the broad inter- and intratumor molecular heterogeneity.

Historically, CRC represented the first model for multistep cancer evolution in which discrete and sequential genetic modifications in specific oncogenes and tumor-suppressor genes occur throughout cancer progression (11, 12). Next Generation Sequencing (NGS) provided significant advances in understanding the molecular basis of CRC (13–15) and indicated that genetic or epigenetic dysregulation of a relevant, but limited, number of molecular pathways typically occurs in human CRC (13, 15, 16). This molecular picture is complicated by the frequent occurrence of individually rare genetic aberrations, which further expand tumor heterogeneity (13–15).

Reflecting the different biology of CRCs, Guinney et al. recently proposed a molecular classification in four consensus molecular subtypes (CMS): CMS1-MSI immune, CMS2-canonical, CMS3-metabolic, and CMS4-mesenchymal (13). Although this might have implications for prognostication and therapy decisions, its immediate transfer to routine diagnostic/clinical settings is seriously challenging in terms of methodology, turnaround time, costs, and mindset. In fact, despite NGS and other omic approaches may disclose a huge amount of molecular details, still very few of them have yet acquired clinical relevance. In example, the use of anti-EGFR therapy is essentially dictated by the RAS (KRAS+NRAS) wild type status, in the clinical routine (17, 18), which however is largely insufficient for the positive selection of responsive patients (19, 20). Treatment with anti-VEGF antibodies is not driven by specific selection criteria due to lack of validated molecular biomarkers (21, 22). Other targeted approaches (i.e., BRAF or PI3K inhibitors used as single agents) failed due to resistance mechanisms (23). These evidences support the need for a paradigm shift in personalized medicine, as suggested by Dienstmann et al. (24): from a one-gene one-drug approach, to a multi-gene multi-drug perspective.

The use of multigene panel sequencing has been recently validated for clinical applications. In example, we introduced a 22 multigene panel sequencing, which includes the clinically relevant RAS and BRAF hotspots, as a routine for the predictive selection of mCRC patients to be subjected to anti-EGFR therapy (25–32). This implementation allowed us to accumulate a large dataset to ask the question of whether application of multigene panel sequencing to the standard diagnostics of mCRC could provide clinically useful information, with no extra-costs in terms of turnaround time and money.

On the basis of results obtained on 639 formalin-fixed and paraffin-embedded (FFPE) tumor samples, here we report that clinical genomic profiling with a multigene panel identifies distinct molecular association patterns (MAPs) and provides great opportunities to unveil co-occurrence of actionable gene mutations to direct more appropriate therapeutic decisions for the majority of mCRC patients.

## Patients and Methods

### Specimen Collection

A total of 779 FFPE tumor samples from mCRC patients were collected from Policlinico Umberto I (Rome, Italy) and from the Department of Public Health, University Federico II, Naples, Italy. The large majority of samples (696/779) were from the primary site, while few (83/779) were from metastatic sites. All samples reached the molecular pathology labs with a medical prescription for determination of RAS/BRAF mutation status for predictive purposes. As such, only scattered clinical-pathological information was available for the two series. For this retrospective observational study all investigations were approved by the Ethics Committee of the University La Sapienza (Prot.: 88/18; RIF.CE:4903, 31-01-2018). All information regarding human material included in the study was managed using anonymous numerical codes, and all samples were handled in compliance with the principles outlined in the declaration of Helsinki. For samples collected at the Department of Public Health, University Federico II, we obtained written informed consent from all patients, in accordance with the general authorization to process personal data for scientific research purposes from “The Italian Data Protection Authority (http://www.garanteprivacy.it/web/guest/home/docweb/-/docwebdisplay/export/2485392).

### DNA Extraction

Tissue samples with a content of tumor-vs.-non-tumor cells below 20% (evaluated at the observation of Hematoxylin and Eosin stained slides) were excluded from the analysis (33). The tumor area was macroscopically dissected to concentrate tumor tissue. Xylene was added once and ethanol was added twice to remove all paraffin from the tissue sample (34). DNA was extracted using QIAamp DNA FFPE Tissue kit (Qiagen GmbH, Hilden, Germany) according to the manufacturer's instructions. Eluted DNA was quantified with Qubit 2.0 Fluorometer (Thermo Fisher Scientific, Van Allen Way, Carlasbad, CA 92008, USA) using Qubit™ dsDNA HS Assay Kit (Thermo-Fisher Scientific, Eugene, Oregon 96492, USA).

### IT-PGM Sequencing and Variant Calling

IT-PGM sequencing was achieved as described (25, 27, 35). Approximately, 10 ng of DNA samples was required to construct barcoded and adaptor-ligated libraries using the Ion AmpliSeq Library kit 2.0 (Thermo Fisher Scientific, Van Allen Way, Carlsbad, CA 92008 USA) and Ion Xpress Barcode Adapter 1-16 Kit (Thermo Fisher Scientific, Van Allen Way, Carlsbad, CA 92008 USA). The samples were analyzed using Ion AmpliSeq Colon and Lung Cancer Research Panel V2 (CLV2, Thermo Fisher Scientific, Guilford, CT 06437, USA) containing a single primer pool to amplify hotspots and targeted regions of 22 cancer genes frequently mutated in CRCs and NSCLCs (29). Templated spheres were prepared using 100 pM of each library using the Ion One Touch 2.0 machine (Thermo Fisher Scientific, Van Allen Way, Carlsbad, CA 92008 USA). Template-positive spheres were loaded into Ion chip 314 or Ion chip 316 and sequenced by IT-PGM machine (Thermo Fisher Scientific, Van Allen Way, Carlsbad, CA 92008 USA). Sequencing data were analyzed with the Ion Torrent Suite (Thermo Fisher Scientific, http://github.com/iontorrent/TS). Variants with a quality <30 were filtered out.

For the purpose of the study, we generated a mutational data set including only samples carrying mutations of established clinical relevance for KRAS (mutations at codon 12, 13, 59, 61, 117, and 146), BRAF (V600E) and PIK3CA (mutations in exon 10 and 21). For TP53, we included in the study samples carrying mutations with defined pathogenic significance according to ClinVar and/or well-established hotspot mutations. We excluded from the study 140 samples carrying variants of unknown clinical significance (VUS) in these genes. For all other genes, we listed all genetic alterations described as pathogenic, likely-pathogenic or predicted deleterious by *in silico* analysis, while benign polymorphisms were not considered.

When appropriate, PolyPhen-2 (**Poly**morphism **Phen**otyping v2; http://genetics.bwh.harvard.edu/pph2/), PROVEAN/SIFT (Sort Intolerant From Tolerant Subsitutions) http://provean.jcvi.org/protein_batch_submit.php?species=human) computational tools were used to predict the possible impact of the detected alterations on the structure and function of the protein (18, 19).

The reference sequence used are: KRAS NM_033360.3, TP53 NM_000546.5, PIK3CA NM_006218.3, BRAF NM_004333.4, NRAS NM_002524.4, FBXW7 NM_033632.3, SMAD4 NM_005359.5, PTEN NM_000314.6, MET NM_001127500.2, STK11 NM_000455.4, EGFR NM_005228.4, CTNNB1 NM_001904.3, AKT1 NM_001014431.1, ERBB2 NM_004448.3, ERBB4 NM_005235.2, FGFR1, NM_001174063.2, ALK NM_004304.4, MAP2K1 NM_002755.3, NOTCH1 NM_017617.4, DDR2 NM_001014796.3, FGFR3 NM_000142.4, FGFR2 NM_000141.4.

### MSI Analysis

Determination of MSI status was investigated on 162 patients (72 of the 639 cases representing the main bulk of the study plus 90 additional cases collected at a later stage and analyzed separately). It was carried out by analysis of BAT25, BAT26, NR21, NR22, and NR24 mononucleotide repeats as previously described (36). Briefly, one PCR primer of each pair was labeled with 1 with either FAM, HEX, or NED fluorescent markers. PCR amplification was performed under the following conditions: denaturation at 94°C for 5 min, 35 cycles of denaturation at 94°C for 30 s, annealing at 55°C for 30 s, and extension at 72°C for 30 s. This was followed by an extension step at 72°C for 7 min. PCR products were run on ABI PRISM 3130xl Genetic Analyzer (16 capillary DNA sequencer, Applied Biosystem). Gene Mapper software 5 (version 5.0, Applied Biosystems, Van Allen Way, Carsvad, CA 92008, USA) was used to calculate the size of each fluorescent PCR product.

### Statistical Analysis

The mutational data set was organized in a matrix composed by 20 columns and 639 rows where each row corresponds to a different sample and each column corresponds to one of 22 different genes whose mutational pattern was characterized. We performed a Principal Component Analysis (PCA) on this mutational dataset in order to classify mutational patterns based on their similarity. Each matrix element M_ij_ (where i is a generic sample and j is a generic gene) can assume the value 0 or 1 if the patient i has no mutation in the gene j or the mutation is present, respectively (37). Each principal component is a linear combination of optimally-weighted original variables, and so it is often possible to ascribe meaning to what the components represent. The statistical analysis was carried out with SPSS statistics or standard R software, version 2.13.1 (http://www.r-project.org).

Statistical analyses on gender, tumor type, tumor location, and MSI-H phenotype were performed on all cases for which appropriate information was available, using both the 639 and the 90 series.

The Pearson's Chi-square test and Fisher's exact test of association was used to determine the relationship between two categories which consist in coexistence of two mutations (pairwise association analysis). A *p* < 0.05 was considered statistically significant.

### TCGA Network Data set

We downloaded gene somatic mutations for 625 patients from the TCGA data portal (https://portal.gdc.cancer.gov/) accessed December 2018 (38, 39). We cleared this dataset from samples carrying VUS, as we did for our dataset (see above). The resulting data set contained 412 patients with their mutational data of the 22 genes included in the CLV2 panel.

We employed the R package TCGAbiolinks (40) to retrieve patient's Microsatellite Instability (MSI) status from the legacy archive of GDC data portal (https://portal.gdc.cancer.gov/).

## Results

### Mutation Profiling of mCRCs, Pairwise Associations, and Identification of Actionable Targets

**Figure 1:**
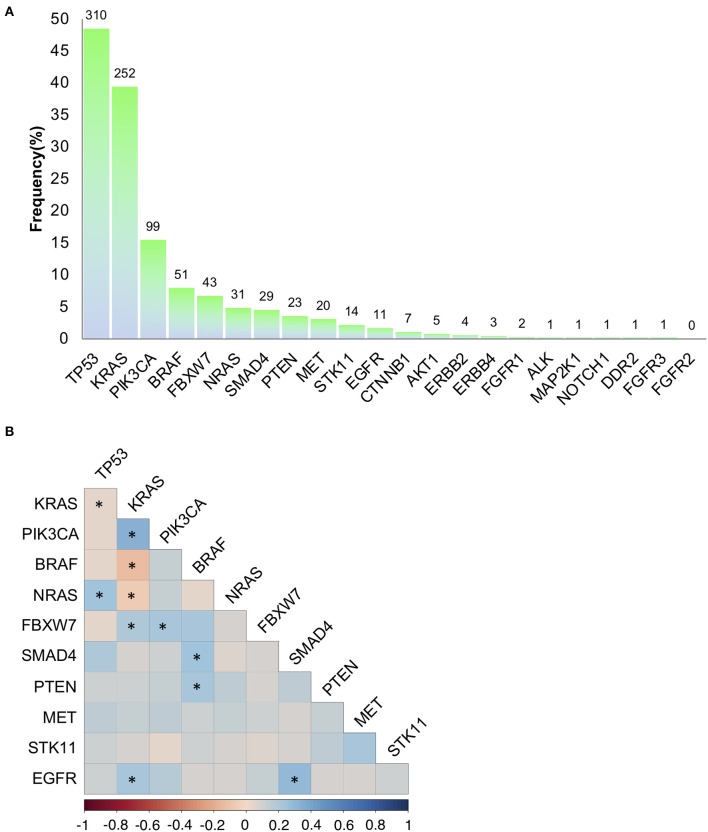
Mutation frequencies and pairwise associations. **(A)** Mutation rates (and absolute numbers of the bars) in 639 metastatic colorectal cancers. **(B)** Correlation Plot describing pairwise association of the mutations occurring on the 11 genes with a mutation frequency >1.5% (mutation number >10). Statistical analysis is given Table 1. *p < 0.05.

Using a 22 gene panel NGS approach, we detected pathogenic mutations in at least one of the 22 targets in 523 out of 639 (81.8%) mCRC samples (Table S1). Mutation spectra and frequencies were in line with previous reports (14, 15, 31) (Figure 1A). Eleven genes displayed a mutation frequency >1.5% (mutation number >10), being *TP53* and *KRAS* the most frequently mutated genes (48.5 and 39.4%, respectively) (Figure 1A). Mutations occurred less frequently (<1.5%) in the other 11 genes (*CTNNB1, AKT1, ERBB2, ERBB4, FGFR1, ALK, MAP2K1, NOTCH1, DDR2, FGFR3*, and *FGFR2*), consistent with the “tail effect” associated with NGS profiling of tumor samples (15) (Figure 1A).

To investigate on mutation associations, we initially performed a pairwise association analysis for those genes with a mutation frequency >1.5%. In agreement with previous literature, *BRAF, KRAS, NRAS* mutations were mutually exclusive, while *PIK3CA* and *FBXW7* mutations frequently occurred in association with *KRAS* mutations. *BRAF* and *SMAD4* mutations were associated, while *TP53* and *KRAS* mutations were negatively associated (Figure 1B, Table 1). We also revealed previously unreported positive association between *EGFR* mutations and *KRAS* and *SMAD4* mutations, while *BRAF* mutations were significantly associated with *PTEN* mutations (Figure 1B, Table 1, Table S2). At variance from *KRAS, NRAS* mutations were significantly associated with *TP53* mutations.

**Table 1:** Significant pairwise associations between most frequent gene mutations.

**Gene^**a**^**	**Status**	**Wt (%)**	**Mut (%)**	***p*^**b**^**	**References**
**KRAS (%)**					
TP53	Mut	200 (51.7)	110 (43.7)	0.047	(44)
PIK3CA	Mut	38 (9.8)	61 (24.2)	<0.001	(43)
BRAF	Mut	50 (12.9)	1 (0.4)	<0.001	(43)
NRAS	Mut	30 (7.8)	1 (0.4)	<0.001	(43)
FBXW7	Mut	20 (5.2)	23 (9.1)	0.05	(31)
EGFR	Mut	3 (0.8)	8 (3.2)	0.03^*^	**New**
**PIK3CA (%)**					
FBXW7	Mut	31 (5.7)	12 (12.1)	0.02	(45)
**BRAF (%)**					
SMAD4	Mut	23 (3.9)	6 (11.8)	0.022^*^	(46)
PTEN	Mut	18 (3.1)	5 (9.8)	0.03^*^	**New**
**EGFR (%)**					
SMAD4	Mut	26 (4.1)	3 (27.3)	0.011^*^	**New**
**TP53 (%)**					
NRAS	Mut	9 (2.7)	22 (7.1)	0.01	**New**

a *The genes with an overall mutational rate higher than 1.5% (number of mutations >10) were considered for statistical analysis*.

b *Chi-squared test*.

* *Fisher exact test*.

Overall, 374/639 (58.5%) patients carried actionable gene mutations, as defined by Chakravarty et al. (41), and 153 patients carried druggable alterations. Importantly, the vast majority of patients positive or negative for specific actionable mutations frequently carried additional relevant genetic alterations (Table 2), which in principle could contribute to an individual variability in patients' responsiveness to standard and target-driven therapies. In example, only 27 (4.2% of the entire series) of the 99 patients carrying *PIK3CA* mutations were *RAS*/*BRAF* WT and only 9 of these (1.4% of the entire series) harbored exclusively *PIK3CA* mutations. On the same line, 17/639 (2.7%) patients carried only *BRAF* mutations, while 34 BRAF mutant samples also carried additional mutations.

**Table 2:** Frequency of co-mutation in genes carrying actionable mutations.

	**Status**	**No. of pts. (%)**	**No. of pts. (%) with additional mutations**
*KRAS*	WT	387 (60.6)	270 (42.2)
	Mut	252 (39.4)	176 (27.5)
*NRAS*	WT	608 (95.2)	491 (76.8)
	Mut	31 (4.8)	26 (4.1)
*BRAF*	WT	588 (92.0)	471 (73.7)
	Mut	51 (8.0)	34 (5.3)
*PIK3CA*	WT	540 (84.5)	423 (66.2)
	Mut	99 (15.5)	89 (13.9)
*EGFR*	WT	629 (98.4)	512 (80.1)
	Mut	10 (1.6)	9 (1.4)
*MET*	WT	633 (99.1)	516 (80.7)
	Mut	6 (0.9)	6 (0.9)
*PTEN*	WT	618 (96.7)	501 (78.4)
	Mut	21 (3.3)	20 (3.1)
*AKT1*	WT	634 (99.2)	517 (80.9)
	Mut	5 (0.8)	4 (0.6)
*ERBB2*	WT	636 (99.5)	519 (81.2)
	Mut	3 (0.5)	3 (0.5)
*ALK*	WT	638 (99.8)	521 (81.5)
	Mut	1 (0.2)	1 (0.2)
*MAP2K1*	WT	638 (99.8)	521 (81.5)
	Mut	1 (0.2)	1 (0.2)

### Identification of Mutational Association Patterns (MAPs)

Although pairwise associations might provide interesting insights into the molecular nature of CRC and represents a step forward in considering the molecular complexity of cancer for prognostic and predictive purposes, we reasoned that a more comprehensive use of the entire mutational profile of each sample could help defining a novel and more precise classification of CRC.

**Figure 2:**
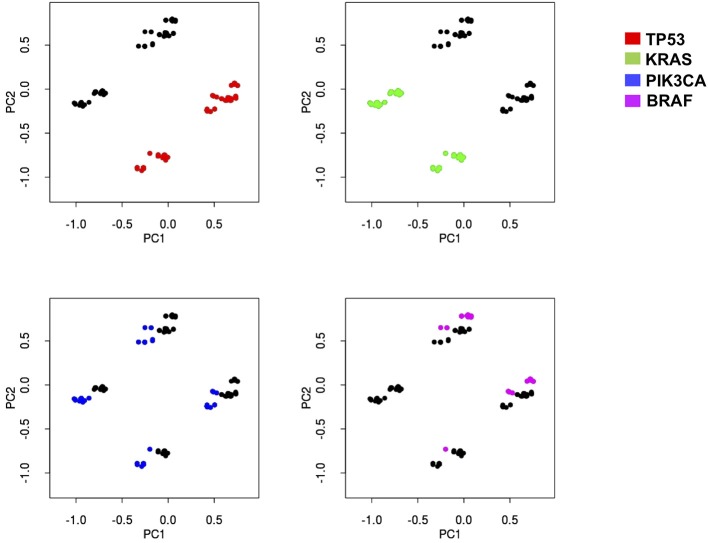
Principal component analysis indicates that four different subsets of mCRC samples may be sharply identified based on KRAS and TP53 mutation status. Principal-component analysis of the sequencing results of 639 mCRCs indicates that the most represented genes in the first two principal components (PC) are able to better separate the data according to their variation. PC1 and PC2 contain 51% of variation in the data. KRAS, TP53, PIK3CA, and BRAF genes have been identified as the most important genes of PC1 and PC2. Each mutational profile has been projected in a two-dimensional space using the PC1 and PC2 to help appreciate sample separation. Each graph indicates how PCA analysis assembles patients (dots) in four distinct groups distinguishable in the two-dimensional space. Red dots, green dots, blue dots, and magenta dots represent samples with mutations in p53, KRAS, PIK3CA, or BRAF, respectively. While KRAS and TP53 mutations sharply map in the four distinct groups in the two dimensional-space, both PIK3CA and BRAF mutations are much less efficient in defining the identity of the four groups, thus indicating that the formers are more effective in creating sharp group separation.

Thus, we subjected our large dataset to a principal component analysis (PCA) with the aim to detect those genes which better classify the different samples based on their overall mutational profile. This approach clearly indicated that two genes (*TP53* and *KRAS*) could sharply cluster our samples into four different subsets: *TP53*^wt^/*KRAS*^wt^ samples, *TP53*^mut^/*KRAS*^wt^ samples, *TP53*^wt^/*KRAS*^mut^ samples, and *TP53*^mut^/*KRAS*^mut^ samples (Figure 2). While mutations in other genes could also aggregate our samples into distinct subsets (see for example *PIK3CA* and *BRAF*, Figure 2), they never reached the sharp effectiveness of *TP53* and *KRAS* mutations.

**Figure 3:**
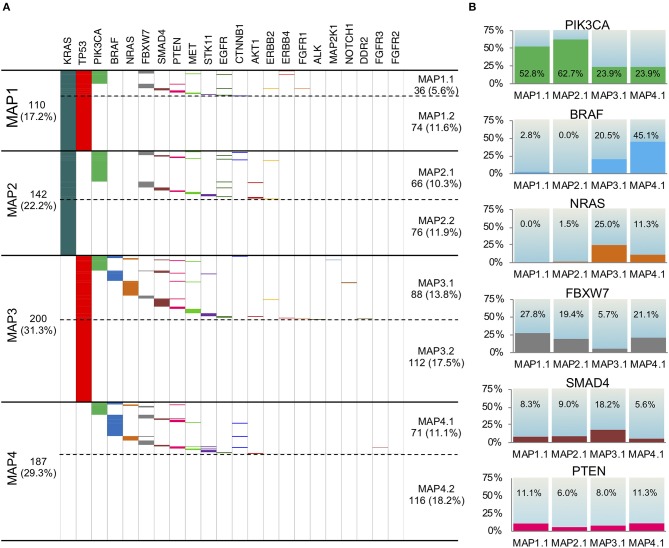
Mutation Association Patterns of the 639 mCRC samples according to the 22 gene panel analysis. **(A)** The presence of a mutation is depicted with a specific color for each gene, while the absence is indicated in white. Four main patterns are obtained, depending on KRAS and TP53 status: MAP1, MAP2, MAP3, and MAP4. Depending on the presence/absence of mutations in genes other than TP53 and KRAS, each MAP could be further divided in two subMAPs (MAP1.1, MAP1.2, MAP 2.1, MAP 2.2, MAP 3.1, MAP 3.2, MAP 4.1, MAP 4.2). **(B)** The frequency of mutation in PIK3CA, BRAF, NRAS, FBXW7, SMAD4, and PTEN genes in the four subMAPs is reported. The Pearson's Chi-square test or Fisher's exact test were carried out and shown in Table S3.

Thus, in accordance to PCA results, we stratified the 639 CRC cases into four different mutation association patterns (MAPs) based on *TP53* and *KRAS* mutation status (Figure 3A). Depending on the presence/absence of mutations in genes other than *TP53* and *KRAS*, each MAP could be further divided in two subsets leading to delineation of a total of eight different MAPs (Figure 3A).

This stratification promptly revealed that 18.2% of the samples carried no mutations in any of the 22 gene of the panel (MAP4.2), while 29.4% harbored only one mutation in either *KRAS* or *TP53* (MAP2.2 and MAP3.2, respectively). An additional 11.6% of the patients only carried *KRAS* and *TP53* mutations with no other alterations (MAP1.2), which indicates that a large fraction of the mCRC cohort is characterized by a very low mutation rate, as detectable by our gene panel sequencing.

The distribution of mutations in genes other than *TP53* and *KRAS* also occurred non-randomly among the MAPs (Figures 3A,B) clearly defining distinct molecular profiles. Indeed, the Pearson's Chi-square test and Fisher's exact test showed statistical significance for almost all the comparisons between the MAPs (Table S3). In details, the eight MAPs are characterized as follows.

*MAP1.1*. This MAP, characterized by the *TP53*^mut^/*KRAS*^mut^ status, showed a high rate of *PIK3CA* mutations (52.8%), rare (2.8%) *BRAF*^V600E^ mutations and no *NRAS* alterations. We also found a relevant number of *FBXW7* mutations (27.8%), and some *PTEN* (11.1%) and *SMAD4* mutations (8.3%), most often mutually exclusive with *PIK3CA* mutations.

*MAP1.2*. This MAP was characterized by the *TP53*^mut^/*KRAS*^mut^ status, and no additional mutations.

*MAP2.1*. This MAP, characterized by *TP53*^wt^/*KRAS*^mut^ status, showed the highest frequency of *PIK3CA* mutations (62.7%). Intriguingly, 3 out of 5 E17K *AKT1* mutations occurred in *PIK3CA* WT samples in this MAP, concurring to the activation of the same pathway.

A fair amount of *FBXW7* mutations (19.4%) and a few *SMAD4* mutations (9.0%), but no *BRAF* and rare *NRAS* mutations (1.5%) occurred in MAP2.1. Coherent with the previously mentioned *KRAS* pairwise association, the rare *EGFR* mutations clustered in MAP1.1 and MAP2.1.

*MAP2.2*. This MAP was characterized by the *TP53*^wt^/*KRAS*^mut^ status, and no additional mutations.

*MAP3.1*. This MAP, characterized by *TP53*^mut^/*KRAS*^wt^ status, had a high frequency of *BRAF* (20.5%), combined with the highest frequency of *NRAS* (in a mutually exclusive way) and *SMAD4* mutations (25.0 and 18.2%, respectively). This group also showed *PIK3CA* mutations in 23.9% of the samples, at least partially non-overlapping with *BRAF, NRAS*, and *SMAD4* mutations, and the lowest frequency of *FBXW7* mutations (5.7%).

*MAP3.2*. This MAP was characterized by the *TP53*^mut^/*KRAS*^wt^ status, and no additional mutations.

*MAP4.1*. In this MAP, characterized by *TP53*^wt^/*KRAS*^wt^ status, we found the highest frequency of *BRAF* mutations (45.1%) and the lowest amount of *SMAD4* mutations (5.6%). It also showed mutations in *PIK3CA, NRAS*, and *FBXW7*, respectively, in 23.9, 11.3, and 21.1% of the samples.

*MAP4.2*. This MAP was characterized by absence of mutations.

Importantly, the analysis of microsatellite instability (MSI) on a test group of 162 samples revealed that 9 tumors were MSI-H. 6 out of 9 MSI-H samples clustered into MAP4.1, 2 in MAP3.1 and 1 in MAP4.2 (Table 3), which suggests that the proposed mutational stratification is able to aggregate samples with similar biology.

**Table 3:** Associations between selected features and MAPs.

				**MAPs**								
		**No**.	**%**	**1.1**	**1.2**	**2.1**	**2.2**	**3.1**	**3.2**	**4.1**	**4.2**	***p***
Gender	M	346	60.1	29 (8.40%)	44 (12.7%)	35 (10.1%)	37 (10.7%)	44 (12.7%)	64 (18.5%)	34 (9.8%)	59 (17.1%)	0.301
	F	230	39.9	15 (6.5%)	37 (16.1%)	28 (12.2%)	26 (11.3%)	41 (17.8%)	33 (14.3%)	22 (9.6%)	28 (12.2%)	
Site	Rectum	89	15.5	9 (10.1%)	14 (15.7%)	17 (19.1%)	5 (5.6%)	9 (10.1%)	13 (14.6%)	6 (6.7%)	16 (18.0%)	0.058
	Colon	486	84.5	35 (7.2%)	67 (13.8%)	46 (9.5%)	58 (11.9%)	76 (15.6%)	84 (17.3)	50 (10.3%)	70 (14.4%)	
Side	Right	183	55.3	12 (6.6%)	31 (16.9%)	23 (12.6%)	18 (9.8%)	36 (19.7%)	15 (8.2%)	34 (18.6%)	14 (7.7%)	**<0.0001**
	Left	148	44.7	12 (8.10%)	17 (11.5%)	15 (10.1%)	19 (12.8%)	24 (16.2%)	36 (24.3%)	3 (2.0%)	22 (14.9%)	
MSI	absent	153	94.4	19 (12.4%)	31 (20.3%)	20 (13.1%)	14 (9.2%)	25 (16.3%)	24 (15.7%)	3 (2.0%)	17 (11.1%)	^*^<0.0001
	present	9	5.6	0 (0.0%)	0 (0.0%)	0 (0.0%)	0 (0.0%)	2 (22.2%)	0 (0.0%)	6 (66.7%)	1 (11.1%)	

* *Fisher's exact test. Bold: statistically significant*.

Mutation distribution of other genes did not vary significantly among MAPs and/or was too low to support major conclusions.

### Identification of MAPs on the TCGA Dataset

To validate MAPs in an external dataset, we accessed the TCGA public mutational data for CRC patients. After appropriate curation of the dataset in order to select all pathogenic mutations potentially identifiable by our multigene panel sequencing approach, we had 412 samples available for MAP stratification. Of interest, the TCGA dataset included all CRC stages, and only a minority of the cases were mCRC (Figure S1A), as already noted by others (14, 42).

The mutation frequencies on the 22 genes included in the CLV2 panel were largely similar between TCGA dataset and our mCRC cohort (Figure S1B). All different MAPs exist with rather similar rates, in TCGA dataset and our series, with MAP 4.1 and MAP4.2 representing sharp exceptions. Indeed, MAP 4.1 accounts for 11.1% of our series of mCRC samples, compared to 18.0% of the TCGA dataset, while MAP4.2 accounts for 18.2% in our series and 5.3% of the TCGA dataset. At variance from our cohort, TCGA dataset included 14% of MSI-H samples, which is consistent with its stage 1-to-stage 4 composition (14). The majority of these cases clustered in MAP4.1, possibly providing an explanation for the different MAP4 rates between the two datasets.

PIK3CA, BRAF, and NRAS mutation rates in the different MAPs display similar trends in our metastatic cohort and in the TCGA dataset (Figures S2A,B). We observed less consistency for the mutation rates of the less frequently mutated FBXW7, SMAD, and PTEN genes.

### Correlation Between Clinical-Pathological Features and MAPs

Next, we examined how MAP stratification correlated with gender and tumor site, the only variables we had available for a reasonable number of patients (Table 3). MAP stratification did not significantly correlate with gender. Concerning tumor site, differences in MAP distribution between colon and rectal localization were close to statistical significance, with a trend for MAP2.1 to be more represented and for MAP2.2 and MAP3.1 to be less represented in rectum compared to colon cancer (Table 3). Moreover, while MAP1 and MAP2 have similar frequencies among right-side and left-side CRC, MAP3.2 and MAP4.2 (accounting for by samples with no mutations or TP53 mutations only) were overrepresented in left-side CRC, and MAP4.1 was overrepresented in right-side CRC. Of relevance, the association of MAP 4.1 with right side remains significant even by omitting MSI-H cases (not shown).

## Discussion

The response of mCRC to current therapeutic approaches is highly variable, reflecting the elevated heterogeneity of the disease (7). This, together with an increasing availability of targeted therapeutic approaches, stresses the need for more comprehensive molecular characterization of each tumor sample, in order to push forward the real achievement of personalized interventions. Despite it is clear that an extended molecular characterization of CRC patients may significantly impact on their clinical management (27–30), very little has entered the clinical routine, yet.

Here, we report that a clinical genomic profiling via multigene panel sequencing allowed identification of pairwise mutation associations and eight distinct MAPs, providing great opportunities to direct more informed therapeutic decisions, in the majority of mCRC cases.

Our data confirm previously reported pairwise gene mutation associations (31, 43–46) and unveil for the first time EGFR/KRAS, EGFR/SMAD4, BRAF/PTEN, and NRAS/TP53 positive associations. The biological or clinical meaning of these associations is difficult to trace, at the moment. In example, while mutations in the EGFR tyrosine-kinase domain are mutually exclusive with KRAS mutations and are positive predictive biomarkers for the efficacy of tyrosine kinase inhibitors in non-small cell lung cancer (NSCLC) (34, 47), these mutations are rare and scarcely relevant in predicting responses to antibody-based anti-EGFR therapy, in mCRC (48). 8/11 EGFR mutation occurring in our mCRC cohort coexist with KRAS mutations. On one side, this may imply that coexisting EGFR and KRAS activating mutation might provide additional advantages to tumor progression in mCRC. This is also supported by the frequent co-occurrence of KRAS and PIK3CA, leading to the constitutive activation of two different pathways downstream of the EGFR. Alternatively, EGFR and KRAS mutations could also exist in different subclonal mCRC populations in the same tumor, as a result of tumor heterogeneity, driving the activation of the same pathway. Addressing the details of tumor heterogeneity and clonality, by tumor multisampling and/or single cell sequencing, will be required to address these issues.

Clinical multigene panel sequencing may easily lead to the identification of actionable and targetable gene mutations (27–31). More importantly, it provides awareness that specific actionable/targetable mutations most frequently co-occur with additional relevant genetic alterations, which in principle could contribute to an individual variability in patients' responsiveness to standard and target-driven therapies. Overlooking this molecular complexity may account for treatment failures, when approved or innovative targeted approaches are used. The scant success of PI3K inhibitors in mCRC may be at least in part due to PIK3CA mutations co-occurring with RAS/BRAF mutations (more than 70% of the PIK3CA^mut^ patients in our cohort) (49). It cannot be excluded that other gene mutations (occurring in an additional 18% of the PIK3CA^mut^ patients in our cohort) may also provide primary resistance to PI3K inhibitors. Only 1.4% of our entire cohort carried exclusively *PIK3CA* mutations, possibly underscoring a subset of patients best suited for treatment with PI3K inhibitors. On the same line, 2.7% (17/639) of the patients carried only *BRAF* mutation, possibly representing the best subset for a target treatment with BRAF^V600E^ inhibitors alone, or in combination with anti-EGFR (50, 51). Most patients carrying BRAF^V600E^ also carried additional mutations, at least some of which might be expected to be involved in primary resistance to anti-BRAF therapies, providing contraindication to single target approaches. In line with this, a phase III 3-arm trial is currently exploring the effectiveness of a triplet therapy with the BRAF inhibitor plus MEK inhibitor associated with the anti EGFR antibody CETUXIMAB in *BRAF*^V600E^ mCRC, in the second or third-line setting (BEACON CRC NCT02928224) (23).

Most importantly, we believe that the greatest added value of clinical multigene panel sequencing may come from a more comprehensive use of the entire mutational profile of each patient to implement a more precise molecular stratification of mCRC. In this observational study, we developed a new stratification system into eight distinct MAPs characterized by non-random, specific mutational combinations. We validated these findings via TCGA data analysis, although few interesting differences emerged. In particular, the different rate of MSI-H cases and the different size of MAP4.1 may be due to the different stage composition between our cohort and TCGA dataset. Whether this is also relevant for the different size of MAP4.2 and the different distribution of some less frequently mutated genes remains to be determined.

**Figure 4:**
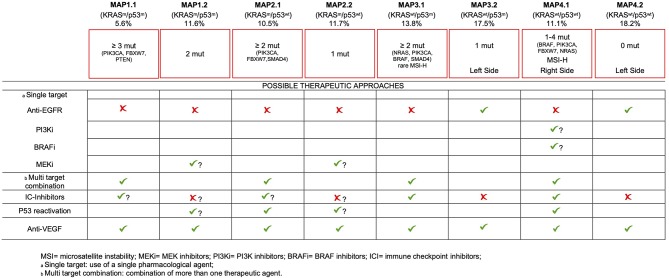
Molecular association pattern taxonomy and potential implications for therapies.

Although we are aware that the lack of clinical data only allows for a speculative proposition, we believe that our comprehensive molecular stratification may provide the base for informed therapeutic decisions, for the majority of mCRC patients, as detailed below (Figure 4).

Firstly, about 50% of our cohort bears no mutations at all (MAP4.2) or just one mutation (MAP2.2 and MAP3.2). An additional 11.6% of patients is characterized by mutations limited to *KRAS* and *TP53* (MAP1.2). Even considering the almost ubiquitous activation of the WNT pathway due to mutations in APC, β-catenin or RNF43 genes (14), these data suggest that the majority of mCRC develop and progress with a low mutation load, which has significant implications for therapeutic interventions. Of interest, the little representation of MAP4.2 in the mixed-stages TCGA dataset compared to our mCRC cohort seems to suggest a higher tendency to cancer progression and a negative prognostic value to having no detectable pathogenic mutations, in addition to being less responsive to common therapies (27). This interesting hypothesis also needs to be confirmed in independent series.

MAP1.1 patients (as well as MAP1.2, MAP2.1, and MAP2.2 patients) are not eligible for anti-EGFR therapy, due to KRAS mutations. More in general, therapies directed against single targets are likely to fail in this group of patients due to primary resistance, as a consequence of having ≥3 gene mutations. Appropriate multitarget combinations should instead be considered for this group, taking advantage of the druggable mutations detected in each patient. Inhibitors of immune-checkpoints are effective in MSS patients (52). The presence of ≥3 pathogenic mutations/tumor in MAP1.1 may suggest a higher mutation rate (compared to MAP 2.2/4.2), raising the possibility to test the efficacy of checkpoint inhibitors, in this subset.

Due to the occurrence of multiple mutations, target driven drug combinations also need to be considered for MAP2.1. However, in this subset we noticed the highest frequency of *PIK3CA* mutations (42/66), 5/66 *PTEN* mutations and 3 out of the 5 *AKT1* mutations, mutually exclusive with *PIK3CA* mutations. Thus, the highest frequency of constitutive activation of the PI3K-AKT1 pathway occurs in this *TP53* WT subset. It has been shown that p53 may limit KRAS dependent transformation (53), suggesting that p53 inactivation may be required for KRAS-dependent cancer development. Nonetheless, KRAS and TP53 mutations are not positively associated in mCRC (31, 44), at variance with NRAS mutations. Of interest, PI3K-AKT axis impinges on MDM2, promoting an increased E3-ubiquitin ligase activity, ultimately leading to p53 functional inactivation via increased degradation (54). Therefore, activation of the PI3K-AKT pathway provides a functional mean to inactivate p53, in KRAS mutant samples. Consistent with this, Singh et colleagues found mutually exclusive occurrence of *TP53* mutations and *PIK3CA* amplification in squamous cell carcinomas (55). This support the possibility that TP53 reactivation approaches, which are being tested elsewhere (56), could also find application in MAP2.1 mCRC (Figure 4).

Beside standard treatment including anti-VEGF, additional intervention is hard to prospect for MAP1.2 patients, since they lack targetable gene mutations, with the possible exception of MEK inhibitors. Mutant TP53 reactivation approaches are yet to come at the clinical level, but they will find potential application also in this mCRC subset. The role of immunotherapy in this subset and in MAP2.2 patients seems counterintuitive, due to the low mutation rate.

Targeting EGFR as a single strategy will probably be ineffective for most patients of MAP3.1 due to the frequent occurrence of PIK3CA, NRAS, BRAF, or SMAD4 mutations, all of which have been related to primary resistance to this approach (17, 43, 57, 58). Therefore, combination treatments should also be carefully planned in this subset. Importantly, few MSI-H patients fall in this group creating opportunities for immune system reactivation therapies.

In sharp contrast, MAP3.2 and MAP4.2 patients, largely coincident with the known “quadruple negative” mCRC subset (24, 59), are probably the most eligible to chemotherapy plus anti-EGFR therapies, since they lack known predictable resistance mechanisms. Of interest, these subsets are prevalent in the left colon, consistent with the observation that TP53 mutations and alternative mechanisms of activation of receptor tyrosine kinase pathways characterize tumors developing in the distal colon (14). These data also fit with the recently reported increased chance of response to anti-EGFR treatment in left colon mCRC (60, 61).

mCRCs of the MAP4.1 subset are predominantly localized to the right colon, where tumors appear to be less responsive to conventional therapies (60, 62). In this subset we detected the highest percentage of BRAF^V600E^ mutant patients, suggesting multiple targeting of BRAF^V600E^ and EGFR, perhaps also in combination with MEK inhibitors (50, 63). Anti-EGFR therapy alone should be possibly avoided, due to the frequent occurrence of primary resistance mutations in PIK3CA, NRAS, BRAF, or FBXW7 (17, 43, 57, 58). TP53 reactivation may also seem reasonable, in cases with NRAS and PIK3CA mutations, similar to MAP2.1 patients. Finally, MAP4.1 also includes the majority of MSI-H mCRC patients, which are most likely to benefit from immune checkpoint inhibitors (52).

Although we are aware that our clinical genomic profiling does not take into account copy number variations and genomic rearrangements that may lead to derangement of specific oncogenic/oncosuppressive pathways, these rarely occur in mCRC (14). It remains that the major limitation of our study is that we had no access to homogeneously collected clinical data, which clearly prevented us from reaching significant clinical conclusion. In example, we cannot infer whether any of the MAPs indicates a better response to anti-VEGF therapy, which is still orphan of biomarkers. Nonetheless, we believe that the simple and cost-effective molecular stratification of mCRC compatible with clinical settings described in this observational study will encourage us and others to design prospective studies to specifically address its effective value for more personalized therapeutic intervention of mCRC patients.

## Data Availability Statement

The original contributions presented in the study are publicly available. This data can be found here: NCBI SRA (https://www.ncbi.nlm.nih.gov/bioproject/PRJNA614492/).

## Ethics Statement

For this retrospective observational studies all investigations were approved by the Ethics Committee of the University La Sapienza (Prot.: 88/18; RIF.CE:4903, 31-01-2018). All information regarding human material included in the study was managed using anonymous numerical codes, and all samples were handled in compliance with the principles outlined in the declaration of Helsinki. For samples collected at the Department of Public Health, University Federico II, we obtained written informed consent from all patients, in accordance with the general authorization to process personal data for scientific research purposes from The Italian Data Protection Authority (http://www.garanteprivacy.it/web/guest/home/docweb/-/docwebdisplay/export/2485392).

## Author Contributions

FB performed NGS and statistical analyses, interpreted the results, and drafted the manuscript. CC recruited samples, collected clinical-pathologic data, and interpreted the results. UM, PPi, and FP recruited samples, performed NGS, and collected sequencing data. DR and EM performed statistical/bioinformatics analyses and interpreted the results. MY and CL performed NGS. PPa, PS, and CB performed statistical analyses. SM, VM, AC, AN, AG, MP, SD, FF, PI, SC, GC, and GT recruited samples and collected clinical-pathologic data. GG conceived, designed, coordinated the study, and drafted the manuscript. All authors reviewed, edited, and approved the manuscript for publication.

## Conflict of Interest

The authors declare that the research was conducted in the absence of any commercial or financial relationships that could be construed as a potential conflict of interest. The reviewer VS declared a past co-authorship with the authors to the handling editor.
