# Supplementary material for: Clinical Multigene Panel Sequencing Identifies Distinct Mutational Association Patterns in Metastatic Colorectal Cancer
Source: Front Oncol. 2020 May 7;10:560. doi: 10.3389/fonc.2020.00560 (PMC7221020; doi:10.3389/fonc.2020.00560)
Supplement: Supplementary file 4 [file Presentation_1.pptx]

## Slide 1
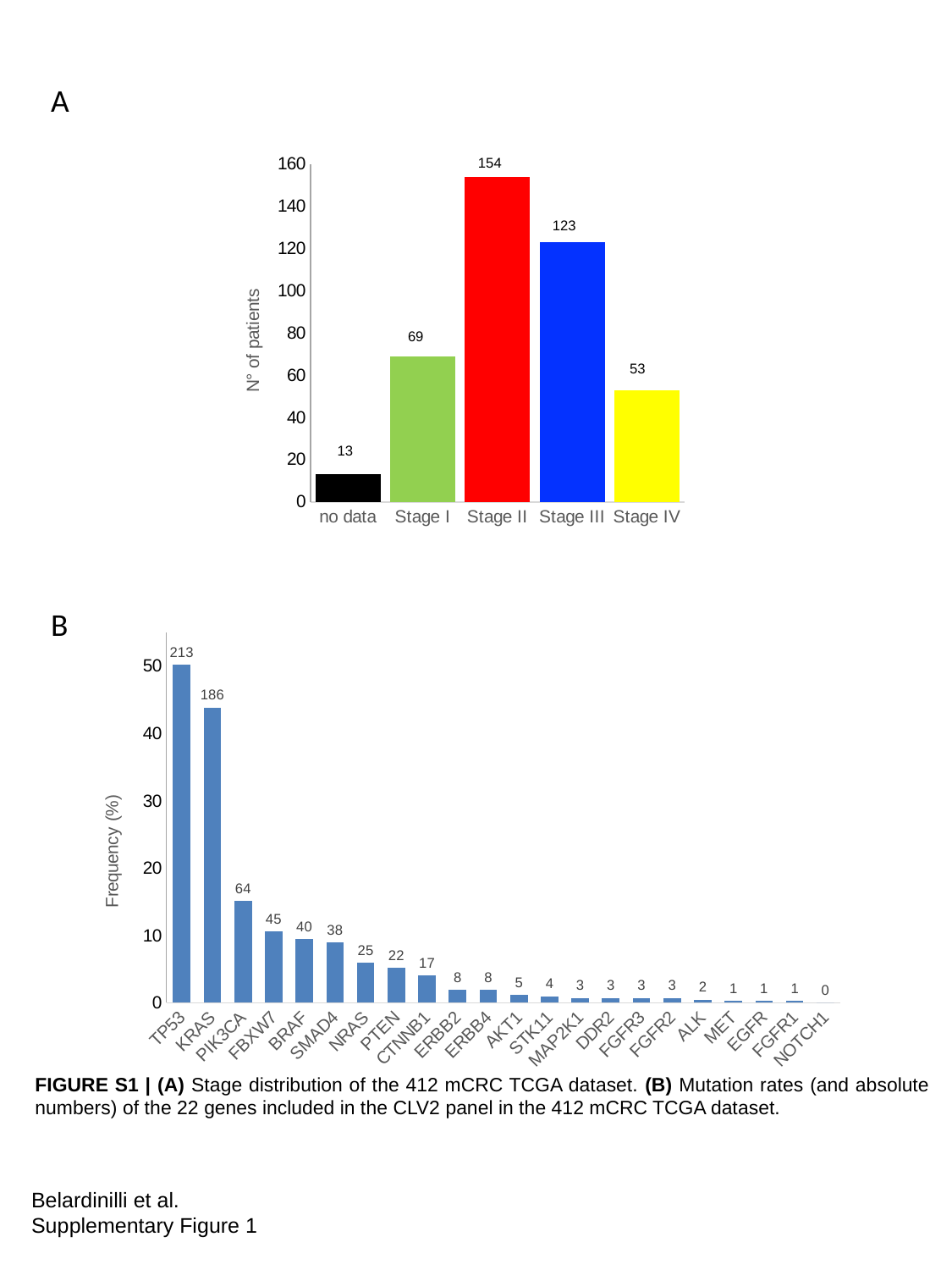

A
### Chart
| Category | |
|---|---|
| no data | 13.0 |
| Stage I | 69.0 |
| Stage II | 154.0 |
| Stage III | 123.0 |
| Stage IV | 53.0 |154
123
69
53
13
B
### Chart
| Category | |
|---|---|
| TP53 | 50.235849056603776 |
| KRAS | 43.86792452830189 |
| PIK3CA | 15.09433962264151 |
| FBXW7 | 10.61320754716981 |
| BRAF | 9.433962264150944 |
| SMAD4 | 8.962264150943396 |
| NRAS | 5.89622641509434 |
| PTEN | 5.188679245283019 |
| CTNNB1 | 4.009433962264151 |
| ERBB2 | 1.8867924528301887 |
| ERBB4 | 1.8867924528301887 |
| AKT1 | 1.179245283018868 |
| STK11 | 0.9433962264150944 |
| MAP2K1 | 0.7075471698113208 |
| DDR2 | 0.7075471698113208 |
| FGFR3 | 0.7075471698113208 |
| FGFR2 | 0.7075471698113208 |
| ALK | 0.4716981132075472 |
| MET | 0.2358490566037736 |
| EGFR | 0.2358490566037736 |
| FGFR1 | 0.2358490566037736 |
| NOTCH1 | 0.0 |FIGURE S1 | (A) Stage distribution of the 412 mCRC TCGA dataset. (B) Mutation rates (and absolute numbers) of the 22 genes included in the CLV2 panel in the 412 mCRC TCGA dataset.
Belardinilli et al.
Supplementary Figure 1

## Slide 2
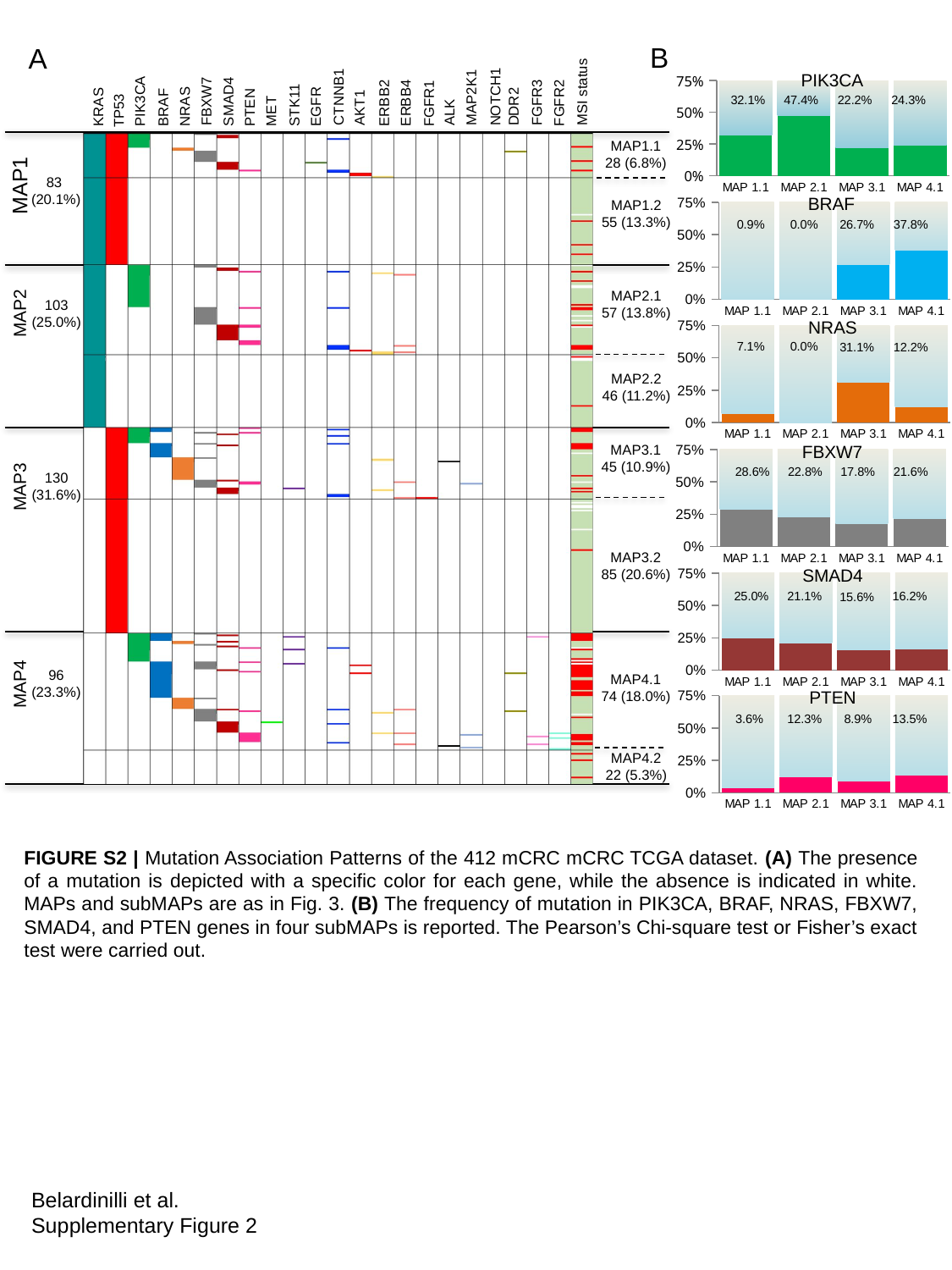

B
A
PIK3CA
### Chart
| Category | | |
|---|---|---|
| MAP 1.1 | 32.1 | 67.9 |
| MAP 2.1 | 47.4 | 52.6 |
| MAP 3.1 | 22.2 | 77.8 |
| MAP 4.1 | 24.3 | 75.7 |MSI status
NOTCH1
CTNNB1
MAP2K1
32.1%
47.4%
22.2%
24.3%
PIK3CA
FBXW7
SMAD4
FGFR3
ERBB4
FGFR2
ERBB2
FGFR1
STK11
NRAS
EGFR
DDR2
KRAS
BRAF
PTEN
AKT1
TP53
MET
ALK
MAP1.1
28 (6.8%)
MAP1
83
(20.1%)
BRAF
MAP1.2
55 (13.3%)
### Chart
| Category | | |
|---|---|---|
| MAP 1.1 | 0.0 | 100.0 |
| MAP 2.1 | 0.0 | 100.0 |
| MAP 3.1 | 26.7 | 73.3 |
| MAP 4.1 | 37.8 | 62.2 |0.9%
0.0%
26.7%
37.8%
MAP2.1
57 (13.8%)
103
(25.0%)
MAP2
NRAS
### Chart
| Category | | |
|---|---|---|
| MAP 1.1 | 7.1 | 92.9 |
| MAP 2.1 | 0.0 | 100.0 |
| MAP 3.1 | 31.1 | 68.9 |
| MAP 4.1 | 12.2 | 87.8 |7.1%
0.0%
31.1%
12.2%
MAP2.2
46 (11.2%)
FBXW7
MAP3.1
45 (10.9%)
### Chart
| Category | | |
|---|---|---|
| MAP 1.1 | 28.6 | 71.4 |
| MAP 2.1 | 22.8 | 77.2 |
| MAP 3.1 | 17.8 | 82.2 |
| MAP 4.1 | 21.6 | 78.4 |28.6%
22.8%
17.8%
21.6%
130
(31.6%)
MAP3
MAP3.2
85 (20.6%)
SMAD4
### Chart
| Category | | |
|---|---|---|
| MAP 1.1 | 25.0 | 75.0 |
| MAP 2.1 | 21.1 | 78.9 |
| MAP 3.1 | 15.6 | 84.4 |
| MAP 4.1 | 16.2 | 83.8 |25.0%
21.1%
16.2%
15.6%
96
(23.3%)
MAP4.1
74 (18.0%)
MAP4
PTEN
### Chart
| Category | | |
|---|---|---|
| MAP 1.1 | 3.6 | 96.4 |
| MAP 2.1 | 12.3 | 87.7 |
| MAP 3.1 | 8.9 | 91.1 |
| MAP 4.1 | 13.5 | 86.5 |8.9%
13.5%
12.3%
3.6%
MAP4.2
22 (5.3%)
FIGURE S2 | Mutation Association Patterns of the 412 mCRC mCRC TCGA dataset. (A) The presence of a mutation is depicted with a specific color for each gene, while the absence is indicated in white. MAPs and subMAPs are as in Fig. 3. (B) The frequency of mutation in PIK3CA, BRAF, NRAS, FBXW7, SMAD4, and PTEN genes in four subMAPs is reported. The Pearson’s Chi-square test or Fisher’s exact test were carried out.
Belardinilli et al.
Supplementary Figure 2
